# The impact of bone marrow irradiation dose on acute haematologic toxicity in cervical cancer patients treated with concurrent chemoradiotherapy

**DOI:** 10.1186/s13014-023-02248-x

**Published:** 2023-04-08

**Authors:** Min Chen, Dajiang Wang, Zhirong Bao, Zongbi Yi, Zijie Mei, Shaoxing Sun, Qingming Xiang, Chunxu Yang, Hui Yang, Hui Qiu, Conghua Xie

**Affiliations:** Department of Radiation and Medical Oncology, Hubei Key Laboratory of Tumor Biological Behaviors, Hubei Cancer Clinical Study Center, Zhongnan Hospital of Wuhan University, Wuhan, 430071 China

**Keywords:** Ddosimetric parameters, Acute haematologic toxicity, Cervical cancer, Concurrent chemoradiotherapy

## Abstract

**Objective:**

To evaluate the impact of bone marrow (BM) irradiation dose on acute haematologic toxicity (HT) in concurrent chemoradiotherapy for cervical cancer.

**Methods:**

Sixty-nine patients with cervical cancer treated with curative or postoperative adjuvant therapy received weekly cisplatin concurrent chemotherapy (CCT) and intensity-modulated radiation therapy (IMRT). The whole pelvic bone marrow (PBM) was delineated and divided into three subsites: ilium (IL), lower pelvis (LP), and lumbosacral spine (LS). Associations between clinical variables, dose volume of BM, including PBM, IL, LP, and LS in the form of x-Vy (volume receiving y Gy for x), and blood cell count nadir were tested using linear regression models. Receiver operating characteristic (ROC) curve analysis was further used to analyse the cutoff values of the variables with *p* < 0.05 in the multivariate analysis.

**Results:**

In 69 patients, the haemoglobin nadir was positive correlated with baseline haemoglobin (*p* < 0.001), negative correlated with relative LP-V10 (*p* = 0.005), relative LP-V25 (*p* = 0.002), relative LP-V50 (*p* = 0.007), relative LP-mean (*p* = 0.003), absolute LP-V15 (*p* = 0.049), absolute LP-V25 (*p* = 0.004) and absolute LP-V30 (*p* = 0.009). The platelet nadir was positive correlated with baseline platelets (*p* = 0.048) and negative correlated with relative LP-V40 (*p* = 0.028), but there was no significant variable in absolute radiation volume by multivariate analysis. No variables related to the neutrophil nadir were found, and the 69 patients were divided into group A (43 cases) receiving 3–4 cycles of CCT and group B (26 cases) receiving 5–6 cycles of CCT. In group A, the relative IL-V15 (*p* = 0.014), the relative IL-V50 (*p* = 0.010) and the absolute LP-V50 (*p* = 0.011) were negative correlated with the neutrophil nadir. No significant variable was found in group B. No significant variables related to the lymphocyte nadir were found, and the neutrophil-to-lymphocyte ratio (NLR) was analysed. Age (*p* < 0.05), relative LP-V15 (*p* = 0.037) and absolute PBM-mean (*p* < 0.001) were found to be negative related to NLR.

**Conclusion:**

The dosimetric parameters of relative irradiated volume of BM have more statistically significant datas on acute HT than absolute irradiated volume. The nadir of haemoglobin and platelets and the vertice of NLR were more affected by the irradiation dose to LP, while neutrophils were more affected by the dose to IL. Acute HT was negative related to both low-dose irradiation (V10-30) and high-dose irradiation (V40, V50). For more than 4 cycles of CCT, the effect of BM irradiation on the neutrophils nadir was masked by chemotherapy.

## Introduction

Concurrent chemoradiotherapy (CCRT), as the standard treatment for locally advanced cervical cancers, has been reported to improve the local control rate and overall survival rate of patients compared with radiotherapy alone [1]. However, acute haematologic toxicity (HT) remains a concern that cannot be ignored, grade 3 or 4 HT was significantly greater in the concomitant chemoradiation group than the radiotherapy group. Such a high incidence of HT often results in prolonged treatment time, increasing the economic burden and compromising clinical outcomes [2]. Therefore, it is important to reduce acute HT.

The high radiosensitivity of bone marrow (BM) haematopoietic cells rich in the pelvis is likely an important contributor to HT. In the clinic, fluorothymidine (FLT) can be used to semiquantitatively calculate the systemic distribution of haematopoietic BM. More than 1/2 of the active haematopoietic BM in the human body is concentrated in the ilium, sacrum, proximal femur and low lumbar spine [3]. When pelvic tumour patients receive radiotherapy, a large amount of functional BM is exposed in the irradiation field, which leads to acute HT [4]. Hence, the association between radiation dosimetric parameters and the occurrence of acute HT needs to be further explored.

Diverse results have been observed regarding the above question. Rose [5] and Albuquerque [6] reported that the volume of pelvic bone marrow (PBM) receiving low-dose radiation such as 10 Gy or 20 Gy is associated with HT among cervical cancer patients undergoing CCRT, while other studies [7] showed that the volume of BM receiving 40 Gy and the mean dose to BM correlated with higher rates of grade ≥ 2 HT. In addition, some studies [8, 9] have suggested that acute HT in cervical cancer patients receiving CCRT is related to both low-dose and high-dose BM irradiation. Despite controversy, interest in dosimetric predictors of acute HT is still growing due to its clinical significance. Efforts have been made to reduce HT, and previous investigations found that intensity-modulated radiation therapy (IMRT) has an advantage in reducing the severity of acute HT by limiting the dose delivered to BM without increasing the toxicity to other normal tissues compared with conventional techniques in pelvic CCRT [10–13]. Therefore, all patients in this study were treated with IMRT.

Furthermore, using the entire bones as a proxy for BM is a limitation, because this includes significant quantities of bone. In our study, the whole PBM was divided into three parts: ilium (IL), lower pelvis (LP), and lumbosacral spine (LS). The effects of irradiation dose on haemoglobin (HGB), platelets (PLT), neutrophils (NEU), lymphocytes (LYM) and neutrophil-to-lymphocyte ratio (NLR), in each part of the PBM were examined. In contrast to previous clinical studies that only evaluated the effect of the relative dose volume of BM irradiation on acute HT, the absolute dose volume was also to be considered in our work. The aim of our study was, therefore, to evaluate the impact of BM irradiation dose on acute HT in pelvic CCRT for cervical cancer during IMRT.

## Methods and materials

### Study population

This study was performed retrospectively, recruiting 69 cervical cancer patients who underwent IMRT and concurrent chemotherapy (CCT) in Zhongnan Hospital, Department of Radiation and Medical Oncology between January 2017 and December 2021. Demographic and clinical information was derived from the electronic medical record system of our institution. The inclusion criteria were as follows: (1) age 18–75 years; (2) pathological diagnosis of cervical cancer (squamous cell carcinoma, adenocarcinoma or adenosquamous carcinoma); (3) FIGO 2018 stage is IB1-IVa, including postoperative adjuvant CCRT and radical CCRT; (4) requirement for concurrent pelvic chemoradiotherapy; and (5) CCT regimen of cisplatin every week with a dose of 40 mg/m^2^. The exclusion criteria were as follows: (1) prior history of pelvic irradiation; (2) neoadjuvant chemotherapy or induction chemotherapy before radiotherapy, which increased acute HT; (3) CCT other than cisplatin; (4) radiotherapy techniques other than IMRT, such as the four-field box technique or Tomo Therapy; (5) treatment with < 3 cycles or > 6 cycles of CCT with cisplatin; (6) extended-field radiotherapy (para-aortic region); (7) incomplete clinical information; and (8) usage of pegylated recombinant human granulocyte colony-stimulating factor during radiotherapy, possibly boosting the blood cell count. A total of 69 patients were finally enrolled in this study.

### RT planning and delivery

All patients assumed a supine position and underwent thin-slice 3 mm computed tomography (CT) scans for simulation. The gross tumour volume (GTV) included the primary cervical tumour and metastatic pelvic lymph nodes with short diameters exceeding 10 mm. The positron emission tomography/computed tomography (PET/CT) or magnetic resonance imaging (MRI) were used for staging and for image fusion in delineation of GTV in inoperable cases. The clinical tumour volume (CTV) consisted of the GTV, cervix, uterus, upper half of the vagina, parametria and pelvic lymphatic drainage areas (common iliac, internal and external iliac, obturator and presacral nodes). For patients with the lower 1/3 of the vagina invaded, inguinal lymph nodes were included. The planning target volume (PTV) was generated by an expansion of 7 mm around draining lymph nodes, 15 mm around the cervix and uterus, and 10 mm around the remaining region of CTV in all dimensions. The small bowel, colon, rectum, bladder and BM were identified and contoured as organs at risk (OARs). Treatment plans were optimized to encompass 95% of the PTV with 100% of the prescribed isodose line, with a hot spot region located in the CTV. The Eclipse treatment planning system version 15.0 (Varian Medical Systems, Palo Alto, CA, USA) was utilized to develop the radiation dose plan. Linear accelerators generating 6-MV photons were adopted to perform EBRT (45–50.4 Gy), after which patients with radical radiotherapy were administered additional brachytherapy treatment using an iridium-192 source once or twice per week with 4 to 5 fractions of 6 to 7 Gy per fraction.

### Chemotherapy delivery

All patients were scheduled to receive weekly cisplatin (40 mg/m^2^) concurrent with EBRT, which was performed under the following conditions: white blood cell count (WBC) > 2.0 × 10^9^/L, absolute neutrophil count > 1.0 × 10^9^/L, HGB > 75 g/dL, PLT > 50 × 10^9^/L, and creatinine clearance > 50 mL/min. Patients were slated to receive three to six cycles of cisplatin during IMRT. Short-acting granulocyte-monocyte colony-stimulating factor and erythropoietin were given under the guidance of oncologists.

### Bone marrow delineation

External contours of bones in the pelvis were delineated in the bone window on planning CT images as an alternative to PBM, which was subsequently subdivided into 3 parts: (1) IL, extending from the iliac crests to the superior border of femoral heads; (2) LP, containing the pubes, ischia, acetabula, and proximal femora, extending from the upper margin of femoral heads to the medical margin of ischial tuberosities; (3) LS, extending from the upper border of the PTV, usually between the 4th and 5th lumbar vertebrae, to the lower part of the coccyx.

Dose volume histogram analyses were carried out to calculate the dose distribution in each BM region. The volume of each region irradiated to more than y Gy was defined as PBM-Vy, IL-Vy, LP-Vy and LS-Vy for PBM, IL, LP and LS, respectively. The volume of each region receiving 5, 10, 15, 20, 25, 30, 35, 40 Gy, 50 Gy and the mean dose were quantified. The relative volume of Vy is defined as R-Vy, and the absolute volume of Vy is defined as A-Vy. The mean dose calculated with relative or absolute volume at site X is defined as R/A-X-mean.

### Haematologic toxicity

Complete blood counts were obtained weekly during CCRT. HT endpoints were referred to as HGB, PLT, NEU, LPM nadirs and NLR vertice occurring during EBRT. HT was graded according to the RTOG acute radiation toxicity scoring criteria [14].

### Statistical analysis

Statistical analysis was performed using SPSS (version 22.0). Linear regression was used to analyse the variables that might affect the nadir of HGB, the nadir of PLT, the nadir of NEU, the nadir of LPM and the vertice of NLR. Age, height, weight, body mass index (BMI), pathological type, radical/postoperative treatment, and the number of CCT cycles were considered confounding variables. The relative or absolute volume of the BM irradiation dose was considered to be the main independent variable and the acute HT including various blood test results which are continuous variables were considered to be dependent variables. Pearson correlation analysis showed a strong correlation between the relative and absolute irradiation volumes (R value > 0.6, *P* value < 0.05). To reduce the influence of collinearity on regression, the relative and absolute volumes of the dose were separately included in the model. First, univariate linear regression analysis was performed for all confounding variables and BM irradiation doses. To include an appropriate number of variables, variables with *P* < 0.08 other than *P* < 0.05 in univariate analysis were selected to be included in multivariate linear regression analysis. Variables with *P* < 0.05 were considered statistically significant in multivariate analysis. If there was no statistical difference in multivariate analysis, subgroups could be divided according to the number of cycles of concurrent chemotherapy, and then further statistical analysis was conducted for subgroups. Receiver operating characteristic (ROC) curves were further used to analyse the statistically significant variables in the multivariate analysis, the value of state variable was 1 which means ≥ grade 2 HT, and the cutoff value at the maximum Youden index was the optimal threshold.

## Results

### Baseline characteristics

The age of 69 cervical cancer patients in this study ranged from 32 to 70 years old, and the average age was 53.4 years; BMI (kg/m^2^) values ranged from 17.7 to 29.2, with an average BMI value of 22.7; the tumour stages of the patients included stage IB-IIIC (67 cases), in addition to 2 cases of pelvic localized recurrence which can be treated by radical radiotherapy; there were 25 cases of radical radiotherapy and 44 cases of postoperative adjuvant radiotherapy; there were 61 cases of squamous cell carcinoma and 8 cases of nonsquamous cell carcinoma; the number of CCT cycles ranged from 3 to 6 cycles; 43 cases (62.3%) involved 3–4 cycles and 26 cases (37.7%) involved 5–6 cycles. The range of HGB values (g/L) before RT was 64–140 with a average of 118.6; the range of PLT (*10^9^G/L) before RT was 98–542 with a average of 252.6; the range of NEU (*10^9^G/L) before RT was 1.7–16.8 with a mean of 4.2; the range of LYM (*10^9^G/L) before RT was 0.6–3.5 with a average of 1.6; and the range of NLR before RT was 0.7–30.6 with a average of 3.3. During radiotherapy, according to the RTOG acute HT classification, there were 54 cases (78.3%) with a grade 0–1 decrease in HGB and 15 cases (21.7%) with a grade 2–4 decrease. Regarding platelets, there were 52 (75.4%) patients with a grade 0–1 decrease and 17 (24.6%) patients with a grade 2–4 decrease in platelets. Regarding neutrophils, there were 34 cases (49.3%) with a grade 0–1 decrease and 35 cases (50.7%) with a grade 2–4 decrease in neutrophils. The above data are shown in Table 1.

**Table 1:** Patient characteristics

Patients (n)		69
Age (years)	Mean (range)	53.4 (32–70)
BMI (kg/m^2^)	Mean (range)	22.7 (17.7–29.2)
Clinical stage (n, %)	IB	23 (33.3%)
	IIA	19 (27.5%)
	IIB	12 (17.4%)
	IIIA	3 (4.3%)
	IIIB	3 (4.3%)
	IIIC	7 (10.1%)
	Recurrent	2 (2.9%)
Type of radiotherapy (n, %)	Radical radiotherapy	25 (36.2%)
	Postoperative adjuvant radiotherapy	44 (63.8%)
Pathological type	Squamous cell carcinoma	61 (88.4%)
	Non-squamous cell carcinoma	8 (11.6%)
CCT cycles	3 cycles	9 (13.0%)
	4 cycles	34(49.3%)
	5 cycles	25 (36.2%)
	6 cycles	1 (1.4%)
Baseline before radiotherapy	HGB mean (range) g/L	118.6 (64–140)
	PLT mean (range) 10^9^G/L	252.6 (98–542)
	NEU mean (range) 10^9^G/L	4.2 (1.7–16.8)
	LYM mean (range) 10^9^G/L	1.6 (0.6–3.5)
	NLR mean (range)	3.3 (0.7–30.6)
Nadir-HGB (n, %)	Grade 0–1	54 (78.3%)
	Grade 2–4	15 (21.7%)
Nadir-PLT (n, %)	Grade 0–1	52 (75.4%)
	Grade 2–4	17 (24.6%)
Nadir-NEU (n, %)	Grade 0–1	34 (49.3%)
	Grade 2–4	35 (50.7%)

### Analysis of variables related to the nadir of haemoglobin

In analysing the variables influencing the nadir of HGB in 69 cervical cancer patients, the results of univariate analysis showed that the variables with *P* < 0.08 included weight, BMI, baseline hemoglobin (B-HGB), R-PBM-V10, et al., as shown in Table 2. Multivariate analysis showed that the nadir of HGB was positive correlated with B-HGB and negative correlated withR-LP-V10, R-LP-V25, R-LP-V50 and R-LP-mean. The cutoff values of these five variables were 96.150 g/l, 74.62%, 44.290%, 7.258% and 2020.850 Gy, respectively, as shown in Table 2. In the multivariate analysis of the absolute dose volume of BM irradiation, the nadir of HGB was positive correlated with B-HGB and negative correlated with A-LP-V15, A-LP-V20 and A-LP-V30. The cutoff values of these four variables were 96.150 g/l, 205.813 cm^3^, 151.947 cm^3^ and 96.740 cm^3^, respectively, as shown in Table 2.

**Table 2:** Multivariate analysis of the nadir of hemoglobin in 69 cervical cancer patients

Variables	*P*	Cut off value	Variables	*P*	Cut off value
Weight	0.182		Weight	0.232	
BMI	0.267		BMI	0.435	
B-HGB	0.000	96.150 g/L	B-HGB	0.000	96.150 g/L
R-PBM-V10	0.859		A-PBM-V20	0.570	
R-PBM-V15	0.597		A-PBM-V25	0.359	
R-PBM-V20	0.801		A-PBM-mean	0.589	
R-PBM-V25	0.546		A-LS-V5	0.371	
R-PBM-mean	0.594		A-LS-V10	0.363	
R-LP-V10	0.005	74.620%	A-LS-V15	0.355	
R-LP-V15	0.922		A-LP-V10	0.202	
R-LP-V20	0.511		A-LP-V15	0.049	205.813 cm^3^
R-LP-V25	0.002	44.290%	A-LP-V20	0.299	
R-LP-V30	0.223		A-LP-V25	0.004	151.947 cm^3^
R-LP-V40	0.983		A-LP-V30	0.009	96.740 cm^3^
R-LP-V50	0.007	7.258%	A-LP-V40	0.839	
R-LP-mean	0.003	2020.850 Gy	A-LP-V50	0.436	
			A-LP-mean	0.084	

### Analysis of variables related to the nadir of platelets

The nadir of platelets of 69 patients with cervical cancer was analysed by linear regression. In univariate analysis, the variables with *P* < 0.08 included BPLT, R-PBM-V50, et al., as shown in Table 3. After multivariate analysis of these variables, it was concluded that the nadir of platelets was positive correlated with baseline platelet (B-PLT) and negative correlated with R-LP-V50, and their cutoff values were 255.5*10^9^ G/L and 17.356%, respectively, as shown in Table 3. The above operation was repeated for the volume of absolute dose of BM irradiation (including PBM, IL, LS, LP), and no factor with a *P* < 0.05 was found in the multivariate analysis as shown in Table 3.

**Table 3:** Multivariate analysis of the nadir of platelet in 69 cervical cancer patients

Variables	*P*	Cut off value	Variables	*P*	Cut off value
Type of radiotherapy	0.384		Type of radiotherapy	0.158	
B-PLT	0.048	255.5*10^9^G/L	B-PLT	0.057	
R-PBM-V50	0.463		A-PBM-V10	0.660	
R-LP-V25	0.738		A-PBM-V15	0.347	
R-LP-V30	0.803		A-PBM-V20	0.277	
R-LP-V35	0.485		A-PBM-V25	0.405	
R-LP-V40	0.028	17.356%	A-PBM-V30	0.445	
R-LP-V50	0.631		A-PBM-V35	0.340	
R-LP-mean	0.887		A-PBM-V40	0.203	
			A-PBM-V50	0.104	
			A-IL-V50	0.259	
			A-LS-V5	0.939	
			A-LS-V10	0.844	
			A-LS-V15	0.464	
			A-LS-V20	0.060	
			A-LS-V25	0.939	
			A-LS-V30	0.999	
			A-LS-V50	0.178	
			A-LP-V25	0.282	
			A-LP-V30	0.405	
			A-LP-V35	0.319	
			A-LP-V40	0.149	
			A-LP-V50	0.126	
			A-LP-mean	0.144	

### Analysis of variables related to the nadir of neutrophils

The variables influencing the nadir neutrophil counts in 69 patients with cervical cancer were analysed by linear regression. In the univariate analysis, the only factor with *P* < 0.08 was age (*P* = 0.056). The absolute dose volume of BM (including PBM, IL, LS, LP) was recalculated, and the only factor with *P* < 0.08 was age (*P* = 0.056).

Considering the neutrophils falling apart and BM dose related, also associated with chemotherapy, while patients are treated first patients enrolled in the study, and not received neoadjuvant chemotherapy or induction chemotherapy, concurrent chemotherapy cycle number less than 3 cycles and is greater than 6 cycles also excluded, as far as possible to ensure the consistency of the baseline of cases was studied, but there are still differences concurrent cycles of chemotherapy. To explore the effect of the number of CCT cycles on myelosuppression, 69 patients were divided into two groups. The patients with 3–4 cycles of CCT were divided into group A (43 cases). Patients with 5–6 cycles of CCT were assigned to group B (26 cases). In group A, the nadir neutrophils occurred on days 8–52 of radiotherapy, and the median time was day 32. In group B, the nadir neutrophils occurred on days 7–45 of radiotherapy, with a median time of day 35. Single-factor linear regression analysis was performed to obtain data with a *P* < 0.08. Multivariate analysis was performed to obtain data with a *P* < 0.08. Two variables with *P* < 0.05 were obtained, namely, R-IL-V15 and R-IL-V50. The cutoff values were found to be 79.2% and 11.7%, as shown in Table 4. The above procedure was also repeated for the absolute dose volume of BM irradiation in group A, and the final multivariate analysis obtained a statistically significant factor, A-LP-V50, whose cutoff value was 30 cm^3^. The above procedure was also repeated in group B. Regardless of whether the relative dose volume or absolute dose volume of BM irradiation was analysed by univariate analysis, only one factor with a significant difference could not be further analysed by multivariate analysis. The only factor with a significant difference was age which was negative correlated with the nadir neutrophils (*P* = 0.026).

**Table 4:** Multivariate analysis of the nadir of neutrophil in 43 patients receiving 3–4 cycles of CCT

Variables	*P*	Cut off value (%)	Variables	*P*	Cut off value
R-PBM-V15	0.237		A-PBM-V50	0.704	
R-PBM-V50	0.921		A-IL-V50	0.794	
R-IL-V15	0.014	79.2	A-IL-mean	0.501	
R-IL-V50	0.010	11.7	A-LS-V50	0.453	
R-IL-mean	0.078		A-LP-V50	0.011	30 cm^3^
R-LS-V50	0.553				
R-LP-V50	0.348				

### Analysis of variables related to the nadir of lymphocytes

The variables influencing the value of nadir LYM in 69 patients with cervical cancer were analysed by linear regression. Both in the univariate analysis of the relative and the absolute dose volume of BM, the only factors with *P* < 0.05 were baseline lymphocyte (B-LYM) (*P* < 0.001) and BMI (*P* = 0.036), while all the dosimetric parameters of BM irradiation were not significantly different.

### Analysis of variables related to the vertice of neutrophil-to-lymphocyte ratio

The vertice of NLR for 69 cervical cancer patients was analysed by linear regression. In univariate analysis, the variables with *P* < 0.08 included age, baseline neutrophil-to-lymphocyte ratio (B-NLR), R-PBM-V5, et al., as shown in Table 5. After multivariate analysis of these variables and ROC curve analysis in which the value of the state variable was taken as the mean value of vertex NLR 15.5, it was concluded that the cutoff values of age (*p* < 0.001) and R-LP-V15 (*p* = 0.037) were 67.5 years and 30 cm^3^, respectively, as shown in Table 5. The above operation was repeated for the absolute dose volume of BM irradiation (including PBM, IL, LS, LP). Age (*p* = 0.001) and A-PBM-mean were identified in the multivariate analysis, the cutoff values of which were 67.5 years and 3040.6 Gy, respectively, as shown in Table 5.

**Table 5:** Multivariate analysis of the vertice of NLR in 69 cervical cancer patients

Variables	*P*	Cut off value	Variables	*P*	Cut off value
Age	0.000	67.5 years	Age	0.001	67.5 years
B-NLR	0.117		B-NLR	0.070	
R-PBM-V5	0.518		A-PBM-V30	0.605	
R-PBM-V10	0.323		A-PBM-V35	0.837	
R-PBM-V15	0.072		A-PBM-mean	0.000	3040.6 Gy
R-PBM-V20	0.752		A-LS-V5	0.312	
R-PBM-V25	0.548		A-LS-V10	0.315	
R-PBM-V30	0.777		A-LS-V15	0.311	
R-PBM-V35	0.948		A-LS-V20	0.334	
R-PBM-V40	0.993		A-LS-V25	0.439	
R-PBM-mean	0.787		A-LS-V30	0.642	
R-LP-V10	0.063		A-LS-V35	0.887	
R-LP-V15	0.037	67.6%	A-LS-V40	0.494	
R-LP-V20	0.323		A-LP-mean	0.293	
R-LP-V25	0.085				
R-LP-V30	0.246				
R-LP-V35	0.534				
R-LP-V40	0.547				
R-LP-mean	0.534				

## Discussion

More attention has been devoted in recent years to identifying the relationships between BM radiation dosimetric parameters and HT in patients undergoing CCRT. MacManus [15] conducted a stratified analysis of acute HT caused by radiotherapy and found that the most important risk factors for treatment interruptions with both thrombocytopenia and neutropenia were CCT and an increasing percentage of marrow irradiation. Mell [16, 17] found in a study of patients with cervical cancer and anal cancer receiving CCRT that acute HT was associated with an increased low dose to PBM. Albuquerque [6] collected 40 patients with cervical cancer undergoing CCRT and conducted multiple logistic regression analysis on the potential predictors of HT, finding that PBM-V20 ≥ 79.42% was an independent predictor of acute HT ≥ grade 2 (r = 0.8, *P* < 0. 0001). Rose [5] established a normal tissue complication probability (NTCP) model of HT in patients with cervical cancer undergoing CCRT. Studies have shown that patients with V10 ≥ 95% and V20 ≥ 76% of BM irradiation are prone to grade 3 or higher HT. Klopp [7] analysed the HT in patients in the RTOG0418 trial and found that in cervical cancer patients who received concurrent IMRT after surgery, when the BM irradiated volume was V40 > 37% or the mean BM irradiated dose was greater than 34.2 Gy, the incidence of grade 2 or higher HT increased. The results of the above studies showed that acute HT was closely related to the radiation volume of BM, especially low-dose radiation such as V10 and V20, and high-dose V40. In our research, we also came to some similar and different conclusions.

The nadir of HGB for 15 cases (21.7%) was grade 2–4, indicating that only a tiny minority of severe decreases in HGB were observed. This study also concluded that the nadir of HGB was correlated with the baseline HGB level before radiotherapy and the dose of BM irradiation, including R-LP-V10, R-LP-V25, R-LP-V50, R-LP-mean, A-LP-V15, A-LP-V20, and A-LP-V30. This indicates that the nadir of HGB during radiotherapy is mainly related to the baseline HGB level before radiotherapy and the dose volume of the LP. Most patients with cervical cancer come to see a doctor because of the symptoms of vaginal bleeding. Due to blood loss, their initial HGB level before treatment is low, and it can be seen from the data in Table 2 that the level ranges from 64 to 140 g/l. On the other hand, compared with the IL and LS, the irradiation dose of the LP has a greater impact on the decrease in HGB. It may be that the LP is the main pelvic haematopoietic bone of HGB, or the HGB haematopoietic function of the LP is more sensitive to radiation. Mell [16] and Zhu [18] found that the strongest associations with HT were the LS and LP, rather than the IL, in cervical cancer patients treated with chemoradiotherapy. The decrease in HGB in this study was associated not only with low-dose radiation but also with high-dose radiation. Wang [9] reached a similar conclusion that PBM dose irradiation of cervical cancer patients treated with CCRT and a process to both low-dose (V16-18) and high-dose (V35, 36 and V47) irradiation was associated with HT, depending on the fractional volumes receiving the variable degree of dosage.

17 cases (24.6%) had grade 2–4 decrease, indicating that the proportion of severe thrombocytopenia was relatively small. We also found that the nadir of platelets was associated with baseline platelet levels before radiotherapy and with the dose of BM irradiation, including R-LP-V40, but not with the absolute dose volume of irradiation. It can be seen from the data in Table 2 that the baseline level of platelets ranged from 98 to 542*10^9^ G/L. This study indicates that in the process of pelvic radiotherapy for cervical cancer, compared with the IL and LS, the irradiation dose to the LP has a greater impact on the decline of platelets, which may be the main pelvic haematopoietic bone of platelets. Lee [19] pointed out that patients with an R-LP-V40 > 23% had higher rates of grade 2 or higher thrombocytopenia (32% vs. 7%, *P* = 0.04) at week 3 in anal cancer. The target volume of anal canal cancer is more downwards than that of cervical cancer, which also results in more radiation exposure to the LP.

The variables of neutropenia in 69 cases of cervical cancer were statistically analysed, and no variables with *P* < 0.05 were obtained. However, according to the different cycles of CCT, the patients were divided into group A with fewer cycles of chemotherapy and group B with more cycles of chemotherapy. Group A clearly had statistically significant data, such as R-IL-V15 and R-IL-V50 in IL and A-LP-V50 in LP. In group B, the radiation dose was not associated with acute HT. This indicates that the decrease in neutrophils was related to the dose of BM irradiation, even if the effect of chemotherapy was not as great. However, after more than 4 cycles of CCT, the effect of BM irradiation dose on neutrophil decline was masked by chemotherapy. Manus’s study [15] reached a similar conclusion that the most important risk factors for treatment interruptions with neutropenia were CCT (OR, 42.1; *P* < 0.001) and increasing percentage of marrow irradiated (OR, 3.3 for each 20%; *P* < 0.001). In conclusion, the decrease in neutrophils is affected by both the number of CCT cycles and the dose of BM irradiation, while chemotherapy may be more important. It is important to note that the relative dose volume to the IL has the greatest effect on acute HT than the dose to the LP and LS, and the absolute dose of the LP-V50 more than 50 Gy was associated with grade 2 or higher neutropenia. In the study of 128 cases of rectal cancer by Cheng [20], PBM and LP dosimetric parameters were correlated with grade 2 or higher neutropenia in the 5FU group but not in the FOLFOX group. It may also be that the chemotherapy scheme of FOLFOX is stronger than 5-FU, and the acute HT caused by chemotherapy is more important, which masks the effect of BM irradiation dose on acute HT. In addition, the median time for the nadir neutrophils in group A was day 32 which is three days earlier than Group B’s day 35. Perhaps because the nadir neutrophils appeared earlier in group A, the subsequent concurrent chemotherapy could not be performed in group A, and the number of cycles of concurrent chemotherapy in group A was less than group B. The decline in LYM was not related to the dose of BM irradiation, but the rate of decline of LYM was faster than that of neutrophils, so NLR showed an increasing trend during CCT. The NLR was associated with the dose of BM irradiation, especially R-LP-V15 and A-PBM-mean in the LP. At present, in cervical cancer, studies have shown that an increased NLR is associated with poor prognosis of cervical cancer [21, 22]. Cheng’s study [23] suggested that high pretreatment NLR values were independently correlated with poor survival in patients with metastatic cervical cancer treated with combination immunotherapy. Therefore, whether controlling the dose of BM irradiation and reducing the NLR can improve the prognosis of patients with cervical cancer needs further study.

But a limitation of this study is its retrospective design. Insufficient sample size is also one of the limitations of this paper. Furthermore, although the samples with too many and too few cycles of concurrent chemotherapy have been discarded, the number of cycles of concurrent chemotherapy is still not completely consistent.

## Conclusions

The relative volume of BM irradiation has more statistically significant datas on acute HT than the absolute volume. The effect of BM irradiation for each part of the PBM on HGB, PLT, NEU, LYM and NLR is not completely the same. The dose to the IL has a greater effect on the decrease in NEU, while the dose to the LP has a greater effect on HGB, PLT and NLR. Moreover, HGB and PLT were more affected by the high dose irradiation, while NLR was more affected by the low dose irradiation, but NEU were affected by both low dose and high dose irradiation. This provides more evidence and practicability for the limitation of BM irradiation dose in IMRT for cervical cancer in the future.

## Data Availability

Please contact author for data requests.

## References

[1] Green JA, Kirwan JM, Tierney JF (2001). Survival and recurrence after concomitant chemotherapy and radiotherapy for cancer of the uterine cervix: a systematic review and meta-analysis. Lancet.

[2] Parker K, Gallop-Evans E, Hanna L (2009). Five years’ experience treating locally advanced cervical cancer with concurrent chemoradiotherapy and high-dose-rate brachytherapy: results from a single institution. Int J Radiat Oncol Biol Phys.

[3] Hayman JA, Callahan JW, Herschtal A (2011). Distribution of proliferating bone marrow in adult cancer patients determined using FLT-PET imaging. Int J Radiat Oncol Biol Phys.

[4] Cao X, Wu X, Frassica D (2011). Irradiation induces bone injury by damaging bone marrow microenvironment for stem cells. Proc Natl Acad Sci USA.

[5] Rose BS, Aydogan B, Liang Y (2011). Normal tissue complication probability modeling of acute hematologic toxicity in cervical cancer patients treated with chemoradiotherapy. Int J Radiat Oncol Biol Phys.

[6] Albuquerque K, Giangreco D, Morrison C (2011). Radiation-related predictors of hematologic toxicity after concurrent chemoradiation for cervical cancer and implications for bone marrow-sparing pelvic IMRT. Int J Radiat Oncol Biol Phys.

[7] Klopp AH, Moughan J, Portelance L (2013). Hematologic toxicity in RTOG 0418: a phase 2 study of postoperative IMRT for gynecologic cancer. Int J Radiat Oncol Biol Phys.

[8] Zhou YM, Freese C, Meier T (2018). The absolute volume of PET-defined, active bone marrow spared predicts for high grade hematologic toxicity in cervical cancer patients undergoing chemoradiation. Clin Transl Oncol.

[9] Wang D, Yin Y, Zhou Q (2022). Dosimetric predictors and Lyman normal tissue complication probability model of hematological toxicity in cervical cancer patients with treated with pelvic irradiation. Med Phys.

[10] Mell LK, Tiryaki H, Ahn KH (2008). Dosimetric comparison of bone marrow-sparing intensity-modulated radiotherapy versus conventional techniques for treatment of cervical cancer. Int J Radiat Oncol Biol Phys.

[11] Hui B, Zhang YB, Shi F (2014). Association between bone marrow dosimetric parameters and acute hematologic toxicity in cervical cancer patients undergoing concurrent chemoradiotherapy: comparison of three-dimensional conformal radiotherapy and intensity-modulated radiation therapy. Int J Gynecol Cancer.

[12] Mell LK, Sirak L, Wei L (2017). bone marrow-sparing intensity modulated radiation therapy with concurrent cisplatin for stage IB-IVA cervical cancer: an international multicenter phase II clinical trial (INTERTECC-2). Int J Radiat Oncol Biol Phys.

[13] Bao ZR, Wang DJ, Chen SP (2019). Optimal dose limitation strategy for bone marrow sparing in intensity-modulated radiotherapy of cervical cancer. Radiat Oncol.

[14] Cox JD, Stetz J, Pajak TF (1995). Toxicity criteria of the Radiation Therapy Oncology Group (RTOG) and the European Organization for Research and Treatment of Cancer (EORTC). Int J Radiat Oncol Biol Phys.

[15] Manus MM, Lamborn K, Khan W (1997). Radiotherapy-associated neutropenia and thrombocytopenia: analysis of risk factors and development of a predictive model. Blood.

[16] Mell LK, Kochanski JD, Roeske JC (2006). Dosimetric predictorsof acute hematologic toxicity in cervical cancer patients treated with concurrent cisplatin and intensity-modulated pelvic radiotherapy. Int J Radiat Oncol Biol Phys.

[17] Mell LK, Schomas DA, Salama JK (2008). Association between bone marrow dosimetric parameters and acute hematologic toxicity in anal cancer patients treated with concurrent chemotherapy and intensity-modulated radiotherapy. Int J Radiat Oncol Biol Phys.

[18] Zhu H, Zakeri K, Vaida F (2015). Longitudinal study of acute haematologic toxicity in cervical cancer patients treated with chemoradiotherapy. J Med Imaging Radiat Oncol.

[19] Lee AY, Golden DW, Bazan JG (2017). Hematologic nadirs during chemoradiation for anal cancer: temporal characterization and dosimetric predictors. Int J Radiat Oncol Biol Phys.

[20] Cheng YK, Ma Y, Zheng J (2019). Impact of chemotherapy regimens on normal tissue complication probability models of acute hematologic toxicity in rectal cancer patients receiving intensity modulated radiation therapy with concurrent chemotherapy from a prospective phase III clinical trial. Front Oncol.

[21] Lee WH, Kim GE, Kim YB (2022). Prognostic factors of dose-response relationship for nodal control in metastatic lymph nodes of cervical cancer patients undergoing definitive radiotherapy with concurrent chemotherapy. J Gynecol Oncol.

[22] Kang SW, Wu JX, Li J (2022). Prognostic significance of clinicopathological factors influencing overall survival and event-free survival of patients with cervical cancer: a systematic review and meta-analysis. Med Sci Monit.

[23] Cheng MX, Li GL, Liu ZA (2022). Pretreatment neutrophil-to-lymphocyte ratio and lactate dehydrogenase predict the prognosis of metastatic cervical cancer treated with combination immunotherapy. J Oncol.

